# Linkage disequilibrium at the APA insecticidal seed protein locus of common bean (*Phaseolus vulgaris *L.)

**DOI:** 10.1186/1471-2229-10-79

**Published:** 2010-04-29

**Authors:** Matthew W Blair, Sergio Prieto, Lucy M Díaz, Héctor F Buendía, César Cardona

**Affiliations:** 1 1/CIAT - International Center for Tropical Agriculture, A. A. 6713, Cali, Colombia

## Abstract

**Background:**

An interesting seed protein family with a role in preventing insect herbivory is the multi-gene, APA family encoding the α-amylase inhibitor, phytohemagglutinin and arcelin proteins of common bean (*Phaseolus vulgaris*). Variability for this gene family exists and has been exploited to breed for insect resistance. For example, the arcelin locus has been successfully transferred from wild to cultivated common bean genotypes to provide resistance against the bruchid species *Zabrotes subfasciatus *although the process has been hampered by a lack of genetic tools for and understanding about the locus. In this study, we analyzed linkage disequilibrium (LD) between microsatellite markers at the APA locus and bruchid resistance in a germplasm survey of 105 resistant and susceptible genotypes and compared this with LD in other parts of the genome.

**Results:**

Microsatellite allele diversity was found to vary with each of the eight APA-linked markers analyzed, and two markers within the APA locus were found to be diagnostic for bruchid resistance or susceptibility and for the different arcelin alleles inherited from the wild accessions. Arc1 was found to provide higher levels of resistance than Arc5 and the markers in the APA locus were highly associated with resistance showing that introgression of this gene-family from wild beans provides resistance in cultivated beans. LD around the APA locus was found to be intermediate compared to other regions of the genome and the highest LD was found within the APA locus itself for example between the markers PV-atct001 and PV-ag004.

**Conclusions:**

We found the APA locus to be an important genetic determinant of bruchid resistance and also found that LD existed mostly within the APA locus but not beyond it. Moderate LD was also found for some other regions of the genome perhaps related to domestication genes. The LD pattern may reflect the introgression of arcelin from the wild into the cultivated background through breeding. LD and association studies for the arcelin gene, linked genes and other members of the APA family are essential for breaking linkage drag while maintaining high levels of bruchid resistance in common bean.

## Background

Common bean (*Phaseolus vulgaris *L.) is an important grain legume crop for the developing world, being a locally produced staple that is critical for income generation and food security [1]. For example, it is the second most important source of protein to the diet in Eastern and Southern Africa and the fourth in Latin America [2]. The crop is affected by a series of disease pathogens and insect pests, among which bruchid (Coleoptera: Bruchidae) species such as *Zabrotes subfasciatus *(Boheman) and *Acanthoscelides obtectus *(Say) are major pests of stored grain [3,4]. For both insects, there are sources of resistance within distant or wild relatives although the latter pest has been harder to control genetically [5]. The mechanism of resistance to these insects has been thought to be based on the insecticidal activities of seed storage proteins produced by the APA (arcelin, phytohemagglutinin and α-amylase inhibitor) gene family which is made up of related genes all located in one region of the common bean genome.

Functionally, the APA proteins are though to provide resistance to bruchids through antibiosis by reducing larval development as well as insect fertility and growth [6,7]. APA proteins vary in their biochemical and physiological properties but have similar expression patterns all being synthesized only in the embryonic axis and cotyledons during seed formation [8] where they can represent from small amounts (under 1%) to large amounts (20 to 30%) of total protein in the mature grain [9-11]. Arcelin differs from other APA proteins in size and electrophoresis pattern [10] and to date, there are seven variants: the first four identified by Osborn et al [12], arcelin 5 found by Lioi and Bollini [13], and two additional variants found by Santino et al. [14] and by Acosta-Gallegos et al. [15] all in wild accessions of common bean. These variants are somewhat similar but different gene copy numbers have been discovered for the variants and each is associated with different levels of resistance to the bruchid insect pest, *Z. subfasciatus *[2,7,16]. Experiments with transgenic transfer of arcelin have raised doubts about the effectiveness of arcelin alone in providing resistance [6,17] however the APA locus is associated with resistance in breeding pedigrees and inheritance studies [18,19] although this can be lost upon backcrossing as in the case of recently-made near isogenic lines for some arcelin alleles [20].

Genetically, the APA gene family is known to consist of a cluster located at a locus on linkage group B4 of the common bean genetic map [21]. Given this position, the APA locus is inherited independently from the locus for another seed protein, phaseolin, which is on linkage group B7 [22]. The APA locus probably evolved from duplication and divergence [23,24] and paralogous gene copies are evident in the sequencing of the APA locus from several arcelin containing wild accessions of common bean [22,24-26].

While arcelin evolved exclusively in the gene pool of wild common beans from Mexico, the α-amylase inhibitors are found more widely within the species. Both arcelin and α-amylase inhibitors are thought to have arisen from a common ancestor, while phytohemagglutinin and arcelin-like genes are found in other *Phaseolus *species and related legumes [24,27,28]. The arcelin locus from wild beans has been introgressed into a range of advanced lines through artificial selection and these are useful stocks for analyzing the APA gene family [29]. Despite these advances, the process of breeding has been hampered by a lack of genetic tools for and understanding about the APA locus.

The objectives of this research were 1) to genetically fingerprint arcelin-containing advanced lines of common bean and their wild progenitors for microsatellites from the APA region of linkage group B4 2) to compare the linkage disequilibrium (LD) around the APA locus in these lines compared to susceptible genotypes from both the Andean and Mesoamerican genepools, and 3) to compare the LD found at the arcelin locus with the LD found across the rest of the genome based on the evaluation of microsatellite markers on all 11 linkage groups of the common bean genome. Since LD is known to vary when calculated for different subpopulations depending on the relatedness of the genotypes within the subpopulation and population structure of the species as a whole [30] we based our LD estimates on the genepool structure of common bean as was done in three recent studies of population structure in the crop [31-33]. In terms of markers, this study focused on arcelin region markers that targeted sequences of the APA gene family [22] or for their genetic proximity to the arcelin locus on linkage group B4 [34,35]; compared to markers from around the genome [36].

## Methods

### Plant Material

A total of 105 genotypes were used for this study including 62 genotypes selected on the basis of their arcelin status and 43 based on previous diversity evaluations [37] as listed in Table 1. The total set included 36 genotypes that were resistant to the bruchid *Z. subfasciatus *and 69 that were susceptible. Included in these were 7 wild accessions of common bean that are the sources of the seven variants of arcelin known to exist and 31 advanced breeding lines from the bruchid resistance program at the International Center for Tropical Agriculture (either RAZ or GG designations). The susceptible genotypes included 24 genotypes which have been used in breeding programs for arcelin resistance and 43 bruchid susceptible common bean genotypes described in Blair et al. [36]. The RAZ lines were developed by backcrossing the Arc1 allele into the red mottled seed class [29]. Seed production for all genotypes was carried out at the International Center for Tropical Agriculture in Colombia.

**Table 1:** Microsatellite allele polymorphism in sources of Arcelin resistance identified in wild bruchid resistant accessions of common bean and in cultivated bruchid resistant and bruchid susceptible breeding lines and varieties.

Source	Arcelin allele	Pedigree	Bruchid resistance^1^	Marker bands (in bp)				
				**BMd15**	**PV-ag004**	**PV-atct001**	**BMd26**	**BMd8**
***Wild beans***								
G12882	Arc1	NA	HR	168	184, 195, 207	196	138	178
G12866	Arc2	NA	I	168	184, 207	200, 204	135	176
G12922	Arc3	NA	S	168	203, 207, 242	196, 200	135	178
G12952	Arc4	NA	HR	168	195, 203, 207, 242	196,200	141	176
G02771	Arc5	NA	HR	168	184, 203, 207	200	141	176
G11051	Arc6	NA	S	168	184, 195, 203, 207	196	NA	176
G24584	Arc7	NA	HR	168	207, 242, 245	200, 208	135	176
***Bruchid resistant Advanced Lines***								
*a) Mesoamerican*								
GG 564-2(3)	Arc5	TALAMANCA///G02771	R	168	207, 242	200	NA	NA
GG 564-2(7)	Arc5	TALAMANCA///G02771	R	168	184, 203, 207	200	141	NA
GG 564-3	Arc5	TALAMANCA///G02771	R	168	184, 203, 207	200	141	176
GG 564-4	Arc5	TALAMANCA///G02771	R	168	184, 203, 207	200	141	178
GG 564-6	Arc5	TALAMANCA///G02771	R	168	184, 203, 207	200	141	176
RAZ 1	Arc1	PVA1025/WI-85-5	HR	168	184, 195, 207	196	141	176
RAZ 4-3	Arc1	859446-67/G 76	HR	168	184, 195, 207	196	NA	178
RAZ 12-1	Arc1	859446-67/G76	HR	168	184, 195, 207	196	141	176
RAZ 15	Arc1	EMP175///859446-67//XAN105	HR	168	184, 195, 207	196	141	178
RAZ 38	Arc1	EX-RICO 23///G12882	HR	168	184, 195, 207	196	138,141	178
RAZ 44	Arc1	EX-RICO 23///G12882	HR	168	184, 195, 207	196	141	176
RAZ 73	Arc1	RAZ12-4/XAN252	HR	168	184, 195, 207	196	138,141	NA
RAZ 75	Arc1	RAZ12-4/XAN252	HR	168	184, 195, 207	196	141	176
RAZ 80	Arc1	RAZ12-4/XAN252	HR	168	184, 195, 207	196	138	178
RAZ 82	Arc1	RAZ12-4/XAN252	HR	168	184, 195, 207	196	138	178
RAZ 85	Arc1	RAZ12-4/XAN252	HR	168	184, 195, 207	196	138	178
RAZ 86	Arc1	RAZ12-4/XAN252	HR	168	184, 195, 207	196	NA	NA
RAZ 87	Arc1	RAZ12-4/XAN252	HR	168	184, 195, 207	196	138	178
RAZ 89	Arc1	RAZ12-4/XAN252	HR	168	184, 195, 207	196	138	178
RAZ 90	Arc1	RAZ12-4/XAN252	HR	168	184, 195, 207	196	138	178
RAZ 91	Arc1	RAZ12-4/XAN252	HR	168	184, 195, 207	196	138	176
RAZ 101	Arc1	RAZ12-4/XAN252	HR	168	184, 195, 207	196	138	178
RAZ 105	Arc1	RAZ24-5/AND885	R	168	184, 195, 207	196	138	178
RAZ 106	Arc1	RAZ 24-5/AND885	HR	168	184, 195, 207	196	138	176
RAZ 163	Arc1	PIJAO///G12882	HR	168	184, 195, 207	196	138	176
*b) Andean*								
RAZ 24	Arc1	859446-67//G76	HR	168	184, 195, 207	196	138	178
RAZ 24-6	Arc1	859446-67//G76	HR	168	184, 195, 207	196	138	178
RAZ 109	Arc1	RAZ 1/CAP3	HR	168	184, 195, 207	196	135	176
RAZ 111	Arc1	RAZ1/AND885	HR	168	184, 195, 207	196	141	176
RAZ 136	Arc1	RAZ1/AND885	HR	168	184, 195, 207	196	141	176
RAZ 138	Arc1	RAZ1/AND885	HR	168	184, 195, 207	196	141	176
***Bruchid susceptible parents***								
*a) Mesoamerican*								
A429	NA	NA	S	152	203, 242	200	141	178
Catrachita	NA	NA	S	152	203, 242	200	141	178
Talamanca	NA	NA	S	152	203, 275	200	141	NA
VAX3	NA	PVPA9576/XAN309	S	152	203, 242	200	141	178
XAN309	NA	SEL986/XAN263	S	152	203, 242	200	141	178
G11360	NA	NA	S	202	203, 242	200	141	NA
G11350	NA	NA	S	202	203, 242	200	141	NA
G14519	NA	NA	S	NA	240, 242	200	141	176
G4825	NA	NA	S	NA	240, 242	200	141	176
DOR364	NA	BAT1215//RAB166/DOR125	S	202	203, 242	200	141	178
BAT477	NA	G3834/G4493//G4792/G5694	S	202	203, 242	200	141	180
G3513	NA	NA	S	202	203, 242	200	141	180
BAT881	NA	G3834/G4025//G3627/G5481	S	NA	203, 242	200	141	180
G21212	NA	NA	S	202	203, 242	200	141	178
DOR390	NA	DOR364/G18251//DOR365/IN101	S	NA	NA	200	141	178
DOR476	NA	DOR367//DOR364/BAT1298	S	202	203, 242	200	141	178
SEL1309	NA	SEL1152///RAB489//A686/G6385	S	202	203, 242	NA	141	178
BAT93	NA	G3709/G1320//G3645/G5478	S	202	203, 242	200	141	178
ICA Pijao	NA	NA	S	202	203, 242	200	141	178
VAX6	NA	NA	S	202	203, 242	200	NA	178
MAR1	NA	BAT85//DC	S	202	203, 242	NA	141	180
J117	NA	NA	S	152	203, 242	200	141	180
Jamapa	NA	NA	S	202	203, 242	200	141	178
G2333	NA	NA	S	152	203, 242	NA	141	178
G855	NA	NA	S	152	203, 242	200	141	180
MAM49	NA	NA	S	152	203, 242	200	141	190
MAM38	NA	A409/DC	S	152	203, 242	200	141	190
G4090	NA	NA	S	202	203, 242	200	141	180
Tio Canela	NA	NA	S	202	203, 242	200	141	178
DOR714	NA	SEL986//DOR476/SEL1235	S	202	203, 242	200	141	178
SEA5	NA	NA	S	202	203, 242	200	141	180
MD23-34	NA	NA	S	202	203, 242	200	141	178
SEA15	NA	NA	S	202	203, 242	200	141	180
G685	NA	NA	S	152	203, 242	200	141	178
SEA21	NA	NA	S	202	203, 242	200	141	180
*b) Andean*								
AFR619	NA	A484 × G4523	S	152	203, 242	200	135	176
Calima (G4494)	NA	NA	S	152	203, 275	200	135	176
CAL143	NA	Bola/AND277	S	152	203, 242	200	135	176
CAL96	NA	Calima2/Argentino1	S	152	203, 275	200	135	176
CIAS-95	NA	NA	S	152	203, 242	200	135	176
EMP122	NA	G4494/SEL97	S	152	203, 242	200	135	NA
EMP178	NA	SEL1405/BAT1366	S	152	203, 242	200	135	176
EMP232	NA	EMP162/EM98/BAT1514	S	152	203, 242	200	141	NA
EMP277	NA	EMP178/PVA800A	S	152	203, 242	200	135	NA
EMP322	NA	PVA3051/EMP178	S	152	203, 242	200	135	NA
EMP355	NA	EMP182/AND635	S	152	203, 242	200	135	176
EMP378	NA	EMP213/EMP224	S	152	203, 242	200	141	NA
G19833	NA	NA	S	152	203, 242	200	135	NA
JB-178	NA	NA	S	152	203, 275	200	135	176
Montcalm (G6416)	NA	NA	S	152	203,242	200	135	176
PC-50 (G18264)	NA	NA	S	152	203, 242	200	135	176
PVA773	NA	G13922//G21721/G6474	S	152	203, 242	200	135	176
PVA800A	NA	G6617//S25084/A21	S	152	203, 242	200	135	176
Quimbaya	NA	NA	S	152	203, 242	200	135	NA
Royal Red	NA	NA	S	152	203, 242	200	135	176
Saladin-97	NA	NA	S	152	203, 242	200	135	176
Velasco Largo	NA	NA	S	152	203, 242	200	135	176
G21657	NA	NA	S	152	203, 242	200	135	NA
G21078	NA	NA	S	152	203, 242	200	NA	178
G21242	NA	NA	S	152	203, 242	200	135	178
G19833	NA	NA	S	152	203, 242	200	135	176
Radical Cerinza	NA	NA	S	152	203,275	200	135	176
Jalo EEP558	NA	NA	S	152	203, 242	200	135	176
BRB191	NA	NA	S	152	203, 275	200	135	176
G19839	NA	NA	S	152	203, 275	200	135	NA
G5273	NA	NA	S	152	203, 242	200	135	178
SEQ1027	NA	NA	S	152	203, 242	200	135	176

^1^HR, highly resistant (0-15% adult emergence); R, resistant (15.1-30% adult emergence; I, intermediate (30.1-50% adult emergence); S, susceptible (> 50% adult emergence).

### DNA extraction

DNA extraction technique was a standard CTAB(2%) and organic-solvent (chloroform-octanol) extraction based on the method of Afanador et al. [37] where 200 μl of extraction buffer (150 mM Tris-HCl, 15 mM EDTA, 1 M NaCl, 1.5% CTAB, 2% β-Mercaptoethanol) was added to 0.5 g of young trifoliate leaf tissue that was ground with a plastic pestle. A further 600 μl of extraction buffer was added and the mixture incubated at 65°C for 45 minutes before adding chloroform:octanol (24:1) mix, shaking for 30 minutes and precipitating the supernatant with 500 μl of isopropanol at -20°C and 125 μl of sodium acetate in a new eppendorf tube. The DNA was pelleted by centrifuging at 12,000 rpm for 10 minutes and cleaned with 500 μl of 70% ethanol. The DNA pellets were then dried and re-suspended in 150 μl of ddH2O to a concentration of 50-100 ng/μl. DNA was then diluted to 10 ng/μl for use as template for the amplification of microsatellites.

### Marker amplification

This study used diversity evaluations for 100 microsatellites, five of which were selected for characterizing the APA region while the rest were based on the results of Blair et al. [34] and were from map locations throughout the genome. PCR conditions for the markers consisted of initial denaturation for 94°C for 5 minutes followed by 30 cycles of 94°C for 1 minutes, 47°C for 1 minute and 72°C for 2 minutes, ending with a final extension period of 72°C for 5 minutes. The microsatellites were run in 4% polyacrilamide (29:1 acrylamide:bisacrylamide) gels at 150 constant watts (1800-2000 volts) and 45°C constant temperature in Biorad Sequi-Gen GT vertical gel rigs for 1.5 hrs. Gels were silver-stained with a re-circulating tank system as described in Blair et al. [36]. Band sizes were determined by comparison to a molecular weight standard based on 10 and 25 bp ladders (Invitrogen, Carlsbad, CA).

### Bruchid resistance screening

Techniques for insect rearing and testing of genotypes for bruchid resistance were similar to those described by Schoonhoven et al. [38]: bruchid resistance was evaluated with 3 repetitions of 30 seeds infested with 6 pairs of *Z. subfasciatus *(Boheman) in a mesh-covered, clear plastic vial (9 cm high × 1.7 cm in diameter) whose walls were covered with sandpaper (No. 150, rough side of sandpaper facing inwards) to avoid egg-laying on the plastic surface rather than the bean seed coat. Experiments were conducted in a rearing chamber at 27°C and 70% RH. Data was collected on number of eggs at 15 days after infestation at which point initial insect parents were removed and on the number of adults emerging before 50 or up to 70 days after infestation. The percentage emergence was calculated based on the total number of adults emerged compared to the number of eggs laid. Genotypes with 0 to 15% total adult emergence were classified as highly resistant (HR), from 15 to 30% as resistant (R), from 30 to 50% as intermediate (I) and from 50 to 100% as susceptible (S).

### Protein extraction and arcelin determination

The presence of the Arcelin protein and the type of arcelin variant were determined according to the methods described by Ma and Bliss [39] where 0.75 g of bean seed flour was dissolved in 250 ul of extraction buffer (1 M NaCl, pH 2.4), vortexed and centrifuged at 14,000 rpm for 15 minutes, the supernatant was transferred and mixed with 50 μl of cracking buffer (100 mM Tris-HCl, 1% SDS, 20% sucrose, 0.05% β-Mercaptoetanol, 0.005% Bromphenol blue) before being vortexed again, boiled for 5 minutes, allowed to cool and centrifuged again for 15 minutes at 14,000 rpm. A 5 μl aliquot of this mixture was loaded onto a stacking 4% polyacrylamide gel in a Biorad Mini Protean 3 Cell gel apparatus that was run at a constant 150 volts until the sample passed into the running gel at which time 25 mA current and 20°C temperature were maintained. Protein gels were stained for 4 to 5 hours in 120 ml of 0.025% Coomassie Blue R-250, 45.4% methanol, 9.2% acetic acid, and 45.4% distilled water then transferred to distaining solutions (I: 10% acetic acid, 50% methanol and II: 7% acetic acid, 50% methanol) for 4 to 5 hours.

### Linkage disequilibrium and association analysis

LD analysis was carried out for the arcelin resistant and susceptible germplasm in Table 1 for the markers described above. LD values for pairs of markers around the APA locus were estimated from the allele data using the software program PowerMarker v. 3.0 [40] with LD measured as the square of the correlation coefficient (r^2^) between alleles at the two loci. LD matrices were visualized by 2-D plot in the same program. LD was calculated for the complete dataset as well as for the sets of resistant wild and cultivated genotypes separately. Given population structure for the cultivated genotypes, these genotypes were separated into Andean and Mesoamerican gene pools for additional evaluation of LD with the same procedure. All LD values were tested for significance with Fisher's exact tests and the P-values were reported. Associations between bruchid resistance traits and genetic markers near or in the APA locus were determined for the genotypes based on a fixed effect linear model in Trait Analysis by aSSociation, Evolution and Linkage (TASSEL) software http://www.maizegenetics.net/tassel. LD estimates were also made for the principal subpopulations for the 105 common bean genotypes from the cultivated Andean and Mesoamerican genepools and from the wild Mesoamerican sources of resistance to bruchids, These subpopulations were confirmed with STRUCTURE [41] analysis was conducted using K = 2 and K = 3 with 50,000 burn-ins and 100,000 iterations using an admixture model in each case.

## Results

### Germplasm survey showed arcelin specific microsatellite alleles

**Figure 1:**
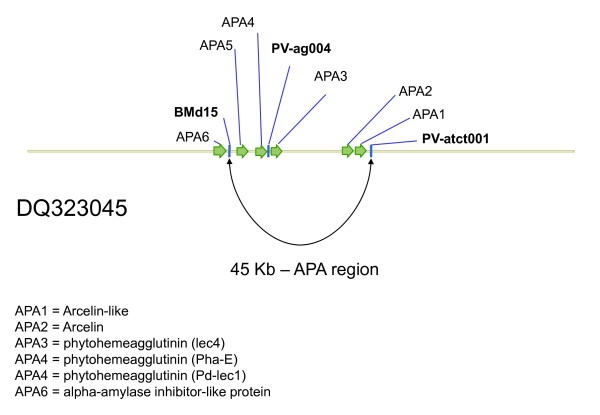
**Schematic diagram of the APA locus showing the placement of arcelin and flanking genes in relationship to SSR markers evaluated for linkage disequilibrium**. Gene annotation for BAC 71F18 based on Kami et al. (2006) and microsatellite markers inidcated in bold.

To begin with, this study focused on some previously developed arcelin region markers selected because they targeted sequences of the APA gene family (BMd9, BMd15, BMd16, PV-ag004 and PV-atct001) as shown in Figure 1 or because of their map location and proximity to the arcelin locus (BMd26, BMd8 and BMd30) on linkage group B4 [34,35]. Among the APA based markers, BMd9 was derived from the gene for D-Lec 2 Phytohemagglutinin-L (Genbank entry X06336), BMd15 from Erythroglutinating Phytohemagglutinin (K03288), BMd16 from Leucoaglutinating Phytohemagglutinin (K03289), PV-ag004 from a Phytohemagglutinin pseudogene (X04660) and PV-atct001 from upstream of the arcelin gene (M68913). BMd9 and BMd16 were found to produce background bands, however all other markers worked well and were evaluated for allelic diversity.

Therefore, with the goal of evaluating the LD around the APA locus and association of this locus with bruchid resistance, the selected microsatellite markers were evaluated in arcelin-containing resistant genotypes including the wild bean sources of the gene, advanced breeding lines with the designations RAZ and GG as well as a corresponding set of bruchid-susceptible genotypes that have been used in breeding programs targeting resistance to *Z. subfasciatus*. The alleles for the five polymorphic and reliable markers are shown in Table 1 with BMd30 as monomorphic and therefore not discussed further.

Microsatellite allele diversity was found to vary with each of the markers tested in the germplasm evaluation. The marker detecting the most alleles was PV-ag004 with 10, while PV-atct001 detected 5 alleles, BMd26 detected 4 alleles, and BMd8 and BMd15 detected 3 alleles each. Within each of the subgroups of germplasm, the diversity observed with the different microsatellites also varied. For example, in the wild accessions that were sources of resistance, BMd15 detected only 1 allele, BMd26 detected 3 alleles, PV-atct001 detected 5 alleles, and PV-ag004 detected 7 alleles. Different banding patterns in the wild accessions were interpreted as separate alleles in the case of PV-ag004 and PV-atct001.

Among the markers, BMd15 was notable in that the allele in the resistant genotypes was different from the alleles in the susceptible genotypes and therefore was predicted to make a good diagnostic marker for bruchid resistance introgressions. The marker PV-ag004 was also diagnostic for the different arcelin alleles and resistant genotypes derived from these despite producing a multiple banding pattern. For example, all the Arc5 containing genotypes except for one had the 184, 203 and 207 bp bands, while all the Arc1 containing genotypes had the 184, 195 and 207 bands and therefore these could generally be distinguished. Meanwhile, all the susceptible genotypes had either of two patterns one with 203 and 242 bp bands and the other with 203 and 275 bp bands. For the PV-atct001 marker Arc5 containing lines could not distinguish from susceptible genotypes however Arc1 containing lines could.

### Distribution of bruchid resistance and association with arcelin linked markers

The results of screening for insect resistance in the germplasm survey are shown in Table 1 and were correlated with the arcelin alleles detected. Among the wild beans, Arc1, Arc4, Arc5 and Arc7 containing accessions had higher resistance (HR) than Arc2, Arc3 and Arc6 containing accessions, which were intermediate (I) or susceptible (S). These results are consistent with those obtained in previous studies by Cardona et al. [29]. All the GG and RAZ series lines with Arc5 or Arc1 alleles respectively were found to be resistant or highly resistant, while all the genotypes that did not contain arcelin alleles both from the Mesoamerican and Andean genepools were found to be susceptible. Interestingly, the Arc1 allele generally provided higher resistance than the Arc5 allele in the cultivated background based on the comparison of the GG series lines versus the RAZ series lines. However this difference was not seen in the wild genotypes with these alleles since G12882 and G02771 had the same levels of bruchid resistance.

Phenotypic data on the cultivated genotypes showed a highly significant association of the markers in the region of the APA locus with bruchid resistance as measured by *Z. subfasciatus *emergence as well as the component trait of number of adults at 50 days after infestation (Table 2). Associations for number of adults at 70 days were significant or close to significance for the markers BMd15, PV-ag004, PV-atct001 and BMd26 while for number of eggs none of the markers were significant. The highest RSq values for number of adults at 70 days were found for the two markers from the arcelin gene itself and the closely associated Phytohemagglutinin pseudogene (PV-atct001 and PV-ag004, respectively). RSq values for this trait were lower for BMd15 and for the markers at greater distances from the arcelin locus such as BMd26 and BMd8, showing a decay in LD across the region in the germplasm analyzed for this study. Meanwhile, the associations for percentage emergence were consistently high across all APA markers.

**Table 2:** Phenotypic variance (RSq) and significance of the association between *Zabrotes subfasciatus *bruchid resistance and markers in the APA region of linkage group B4 of common bean given a linear model analysis of variance with population structure as covariate.

Trait	BMd15	PV-ag004	PV-atct001	BMd26	BMd08
Number of Eggs	0.1716^ns^	0.1933^ns^	0.1892^ns^	0.1317^ns^	0.2313^ns^
Number of Adults (50 d)	0.803***	0.804***	0.804***	0.798***	0.857***
Number of Adults (70 d)	0.229**	0.404**	0.373***	0.189^P = 0.06^	0.147^ns^
Total Adults	0.793***	0.755***	0.750***	0.558**	0.402^ns^

Percentage Emergence	0.923***	0.965***	0.954***	0.976***	0.908***

Significance (P-values): ns = not-significant, *<0.05, **<0.01, ***<0.001

The high values and high significance of the RSq values for the association of markers from linkage group B4 with the bruchid resistance traits was due in part to the highly contrasting averages for adults at 50 days and percentage emergence among the resistant wild or cultivated genotypes versus the susceptible genotypes (Table 3). The number of adults at 50 days averaged 5.0 and 2.9 for the wild and cultivated resistant genotypes, respectively, and 86.8 for the susceptible genotypes.

**Table 3:** Averages and standard deviations for bruchid resistance traits in the different germplasm groups tested.

Germplasm Group	Eggs laid	Adults at 50 d	Adults at 70 d	Total Adults	% Emergence
Resistant wild	48.0 ± 26.9	5.0 ± 4.0	2.9 ± 2.9	7.9 ± 6.2	16.4 ± 12.1
Resistant cultivated	94.7 ± 36.3	2.9 ± 2.8	2.0 ± 2.5	4.8 ± 4.5	5.0 ± 4.2
Susceptible cultivated	93.3 ± 33.3	86.8 ± 31.4	0.3 ± 1.4	87.1 ± 31.3	93.8 ± 4.8

The wild beans were more variable than the resistant breeding lines perhaps given the multiple alleles evaluated in the wild (7) versus the alleles incorporated into RAZ or GG lines (2). More adults emerged at 70 days for the resistant genotypes than for the susceptible genotypes due to the slow development of the insects in the resistant lines. In other words, for the susceptible genotypes the seeds were already completely perforated and consumed so no more adults emerged in the period from 50 to 70 days, while in the resistant genotypes few adults emerged either at 50 or 70 days. As a result percentage emergence was highly contrasting between wild and cultivated resistant genotypes (16.4 and 5.0%, respectively) versus the susceptible genotypes (93.8%).

**Figure 2:**
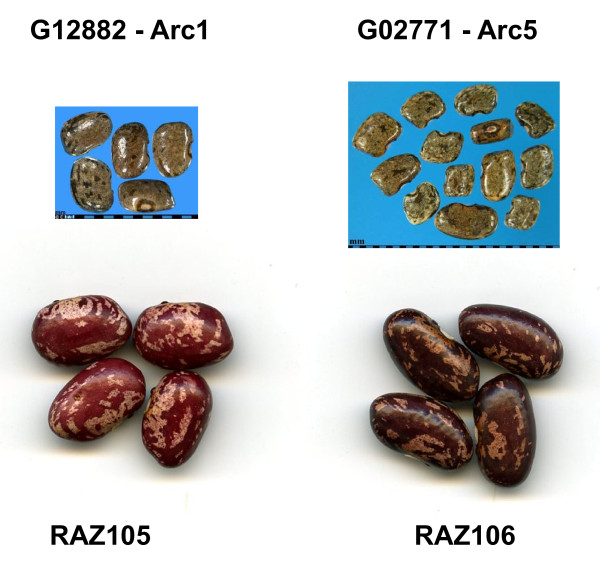
**Relative size of bruchid-resistant wild and cultivated beans used in the study**. Wild bean contain the Arc1 and Arc5 alleles, respectively, and the cultivated beans are two RAZ lines containing arcelin-based bruchid resistance introgressed into a cultivated background.

The higher percent emergence among the wild resistant genotypes can be explained by the variability in the different arcelin/APA alleles which led to some variability for resistance with some genotypes being intermediate (Arc2) or even susceptible (Arc3 and Arc6) in our assay although Arc1, Arc4, Arc5 and Arc7 were highly resistant. One interesting point is that fewer eggs were laid on the wild beans (48.0) than on the cultivated beans (94.0 on average). This is likely to have occurred because the wild beans are smaller seeded and therefore can host fewer eggs than the large seeded cultivated beans. Figure 2 shows the relative size of the wild beans containing the Arc1 and Arc5 alleles and two RAZ lines with arcelin-based, bruchid resistance introgressed into a cultivated red mottled background. It is notable that egg laying is similar on the cultivated resistant and cultivated susceptible genotypes since arcelin does not affect antibiosis at the level of oviposition but rather during larval development [2].

In terms of trait correlations, no significant correlations were observed between egg laying by *Z. subfasciatus *and total number of adults or adult emergence across all the genotypes (Table 4). However, when correlations were estimated for the resistant cultivated and wild genotypes alone they were significant. Meanwhile, the correlation values between adults at 50 and 70 days was only significant with the resistant cultivated genotypes, where emergence of adults was slower than in susceptible cultivated genotypes, as discussed above. Finally, percentage emergence was also correlated with total number of adults in the resistant cultivated genotypes and the r-values were high for this correlation in the resistant wild genotypes as well.

**Table 4:** Correlations (r-values) between bruchid resistance traits in wild and cultivated resistant or susceptible common bean genotypes.

Germplasm Group	Eggs vs. Adult total	Eggs vs. emergence	Adults at 50 vs.70 days	Adult total vs. emergence
Resistant wild	-0.221^ns^	-0.854*	0.613^ns^	0.644^ns^
Resistant cultivated	0.434*	0.062^ns^	0.507**	0.858**
Susceptible cultivated	0.356^ns^	-0.112^ns^	-0.012^ns^	0.001^ns^
All Genotypes	0.243^ns^	0.004^ns^	-0.360*	0.380*

Significance (P-values): ns = not-significant, *<0.05, **<0.01

### Linkage Disequilibrium near the APA locus and throughout the bean genome

Linkage disequilibrium was evaluated between all the markers tested in the germplasm survey based on the pattern of diversity of alleles of each of the microsatellites and map location according to Blair et al. [34]. We were especially interested in confirming that unique alleles most closely associated with the presence of arcelin were not present in any susceptible genotypes and in studying the decay of LD between markers near the APA locus compared to the distribution of LD in other regions of the genome or on other linkage groups. Linkage disequilibrium as measured across all the genotypes in the germplasm survey described above was moderate for the microsatellite markers that were closely linked to the arcelin gene (BMd15, PV-ag004 and PV-atct001) while the two markers that were located further away (BMd8 and BMd26) showed less LD (Table 5). Within the locus the LD values ranged from r^2 ^of 0.5390 to 0.6495, while across the adjacent interval the LD values ranged from r^2 ^of 0.0475 to 0.3966.

**Table 5:** Linkage disequilibrium (r^2^) of microsatellite markers at the APA locus and in flanking regions in all genotypes and in subpopulations of wild resistant, cultivated resistant and cultivated susceptible genotypes.

Marker	BMd15	PV-ag004	PV-atct001	BMd26	BMd8
**Genetic distance (cM)^1^**	**0**	**8**	**10**	**35**	**38**

**All genotypes**					
BMd 15	1.0	0.6091***	0.5390***	0.3212***	0.0475**
PV-ag004	-	1.0	0.6495***	0.3966***	0.1481***
PV-atct001	-	-	1.0	0.3801***	0.1037***
BMd26	-	-	-	1.0	0.2488***
BMd8	-	-	-	-	1.0
					
**Andean genepool**					
BMd 15	1.0	0.1156***	0.3681***	0.0911***	0.0187^ns^
PV-ag004	-	1.0	0.2894***	0.1270***	0.1159***
PV-atct001	-	-	1.0	0.3154***	0.0838**
BMd26	-	-	-	1.0	0.2696***
BMd8	-	-	-	-	1.0
					
**Mesoamerican genepool**					
BMd 15	1.0	0.4459***	0.4171***	0.2444***	0.0683**
PV-ag004	-	1.0	0.5252***	0.5473***	0.6560^ns^
PV-atct001	-	-	1.0	0.5855***	0.0398^ns^
BMd26	-	-	-	1.0	0.0406^ns^
BMd8	-	-	-	-	1.0
					
**Wild**					
PV-ag004	NA	1.0	0.5553^ns^	0.7216**	0.4703*
PV-atct001	-	-	1.0	0.3253^ns^	0.1293^ns^
BMd26	-	-	-	1.0	0.3863^P = 0.09^
BMd8	-	-	-	-	1.0
					
**Cultivated resistant**					
PV-ag004	NA	1.0	0.9163***	0.2006**	0.1172^ns^
PV-atct001	-	-	1.0	0.2006***	0.1172^P = 0.09^
BMd26	-	-	-	1.0	0.3083***
BMd8	-	-	-	-	1.0

1/Genetic distances according to Blair et al. (2003) with mapping of BMd8 from Ochoa et al. (2006). Significance of LD evaluated with Fisher's exact tests, P-values: *<P = 0.05; **<0.01; ***<0.001; ns, not-significant, NA = not applicable, no LD test applied to marker combinations where one marker had no allelic diversity in the subgroup

**Figure 3:**
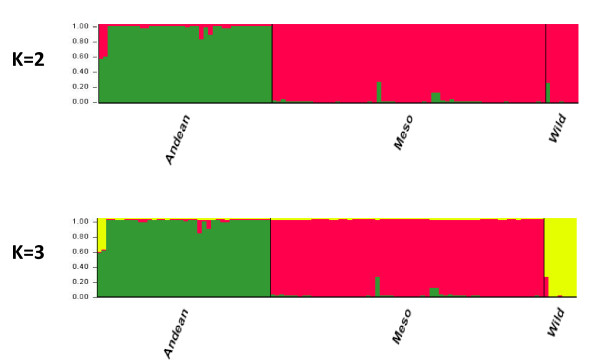
**Population structure of Andean and Mesoamerican, bruchid-susceptible and resistant beans used in the study**. Two levels of subdivision, K = 2 and K = 3, are considered for the 105 common bean genotypes from the cultivated Andean and Mesoamerican genepools and from the wild sources of resistance to bruchids.

Part of the observed LD would be explained by population structure since we included both Andean and Mesoamerican beans in the overall germplasm survey and because we included genotypes bred intentionally for bruchid resistance as well as susceptible genotypes. Therefore, we subdivided the survey into sub-populations based on STRUCTURE analysis finding separation of groups based on gene pool identity at K = 2 and the wild beans diverging at K = 3 as shown in Figure 3. This was significant since the Andean and Mesoamerican genepools are the major source of population structure in common beans but wild beans are very diverse [31-33].

By doing this we found that LD at the APA locus was lower in Andean genotypes than in the Mesoamerican genotypes. Meanwhile LD values between the two APA associated markers, PV-atct001 and PV-ag004, remained high in the cultivated resistant and wild resistant genotypes with r^2 ^of 0.9163 and 0.5553, respectively. Indeed, while the allele for BMd15 found in the wild sources was unique to the wild accessions and the derived introgression lines (RAZ or GG designation) and not present in any of the susceptible lines; the alleles of the markers BMd26 and BMd8, which map further away from the arcelin locus according to Blair et al. [34], presented a much lower association with the arcelin introgression or LD with the APA locus markers PV-ag004 and PV-atct001 in the cultivated resistant lines. For example, LD values between the APA locus markers and the markers BMd26 and BMd8 ranged from r^2 ^of 0.1172 to 0.2006 for cultivated resistant genotypes and r^2 ^of 0.0202 to 0.0990 for cultivated susceptible genotypes.

**Figure 4:**
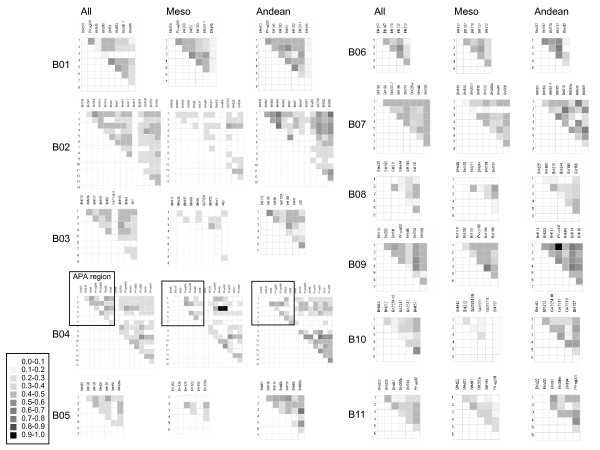
**Allele pair linkage disequilibrium matrices for each linkage group across the entire common bean genome for the full group of genotypes and for the Andean and Mesoamerican group genotypes**. Linkage groups are indicated as B1 through B11 based on map order from Blair et al. (2003). Darker squares indicate higher linkage disequilibrium in terms of correlation coefficient (r^2^) as indicated in the legend. B04, the linkage group with the APA locus is highlighted. Genepools are abbreviated as Mesoamerican (Meso), Andean (Andean) and both together (All).

LD values with APA locus markers were compared with those from around the common bean genome and are shown in Figure 4. LD values were averaged per linkage group and ranged from r^2 ^of 0.1425 (for B3) to 0.4512 (B9) when considering only Mesoamerican genotypes and from r^2 ^of 0.2720 (B8) to 0.6590 (B9) when considering only Andean genotypes. Apart from B9, high average LD was also observed for Mesoamerican genotypes on linkage groups B1, B6 and B7 (all with average r^2 ^above 0.3) and for Andean genotypes on B6, B7 and B11 (all with average r^2 ^above 0.45). It was notable that LD values across the genome were slightly higher when considering the Andean genotypes than when considering the Mesoamerican genotypes.

## Discussion

### The germplasm survey revealed varying levels of marker diversity

As part of the process to evaluate the microsatellite markers associated with APA genes we carried out a germplasm survey to determine the allelic diversity for the markers. In this part of the study, we found the number of alleles and level of polymorphism present for each of the eight microsatellites evaluated to vary substantially. Two microsatellites were found to be clearly associated with the presence of arcelin and the arcelin alleles in the germplasm survey. Of these, the microsatellite PV-ag004 was more polymorphic than PV-atct001. For both markers, unique alleles were found for the sources of arcelin compared to susceptible germplasm resulting in diagnostic tests for Arc1 and Arc5, the most commonly introgressed alleles of the arcelin gene [19].

Several microsatellites, notably PV-ag004 and PV-atct001, produced multiple banding, patterns especially in the wild beans which was interesting because these arcelin and phytohemagglutinin-based markers are part of sequences in the APA gene family which has several highly similar multiple copy sequences at least for the Arc5 [22]. It is interesting to note that microsatellites have been discovered in or near the arcelin and phytohemagglutanin members of the APA family, but none have been found in the alpha amylase gene. Additional multi-copy microsatellites have been found within a bacterial artificial chromosome clone from the Arc4 locus (pers. communic F. Sparvoli).

### Linkage disequilibrium at the APA locus versus other regions of the genome

Given the large number of repeated sequences within the region of the APA locus we suspected LD might be low within this region however it was moderate, although it varied near and away from the region. For example among the markers PV-ag004 and PV-atct001 LD was high but then decayed towards markers BMd26 and BMd8. We found it interesting to contrast that decay of LD across these two groups of markers in wild versus cultivated Andean and Mesoamerican germplasm.

When wild and cultivated genotypes were evaluated separately, LD was relatively high in the wild genotypes both among the APA markers (r^2 ^= 0.4638) and across APA and non-APA markers from the same linkage group region (r^2 ^= 0.2971 to 0.7216). In contrast, for the resistant cultivated lines, LD was high between the two markers (r^2 ^= 0.9163 for PV-ag004 and PV-atct001) but low between these markers and the markers BMd26 and BMd8 (r^2 ^from 0.1172 and 0.1911). This may reflect reduced recombination within the segment containing the APA locus due to introgression from the wild accessions into the cultivated background. The pedigrees of the cultivated lines containing the arcelin introgression generally involved from one to two backcrosses with a standard Andean (eg. G76) or Mesoamerican (eg. Talamanca) background although some early lines in the Andean group were from simple crosses and some later lines were from crosses of a fixed RAZ line with another advanced line such as XAN252, CAP3 or AND685. The wild beans had high LD in contrast to the results of Rossi et al. [33] perhaps because our wild beans were all of Mesoamerican origin as this was the source of the arcelin locus.

Furthermore, LD for the APA locus was lower in the Andean genepool than in the Mesoamerican genepool. This difference may be due to the effect of introgressing the APA locus into an Andean genetic background compared to a Mesoamerican background given that the source of the arcelin gene is in wild Mesoamerican beans. The higher LD within the APA locus than outside it suggests that the APA locus from the wild sources of arcelin is often inherited as a block when it is backcrossed into a cultivated genetic background. This may explain why linkage drag has been observed in the breeding program for bruchid resistance, where it is possible to obtain advanced lines with resistance from the wild sources but these tend to have associated deleterious characteristics, such as lower yield potential and poor architecture which are associated with wild accessions of common bean.

The arcelin locus was unique and interesting for this study because of the microsatellites embedded near the APA family members and because of its status as both a seed storage protein locus and for its involvement in preventing insect herbivory. In contrast to the region of the APA locus, average LD values were high in several regions of the genome including linkage groups B6, B7 and B9 for both genepools and B1 for Mesoamerican beans and B11 for Andean beans. High LD in these regions could be explained by reduced diversity on certain linkage groups associated with the domestication genes *fin *and *ppd *on B1 [42] or heavily selected genes for seed color and seed size, *p *and *Phs *on B7; while the high average LD on B9 could be due to the effect of *Fr*, a fertility restorer gene on this linkage group.

Linkage disequilibrium between alleles at different loci varies with different plant genomes depending on mating system and location within the genome; with self pollinating species generally having higher levels of LD than cross-pollinating species and certain regions having higher LD due to reduced recombination or effects of selection [30]. In this sense, common bean as an inbreeding species should have a lower level of LD decay than outcrossing species although this would vary for different subpopulations of wild and cultivated genotypes [33].

Variability in recombination can be due to chromosomal context and structure, so that greater crossing-over occurs in euchromatin segments in gene dense regions, compared to heterochromatin or gene-poor regions [43,44]. Furthermore, recombination is generally higher within tandemly repeated gene families especially those at disease resistance gene analog loci [45] and perhaps at seed storage protein loci [46]. In this context, the findings of this study are interesting because the moderate LD found at the APA locus may reflect the levels of recombination within the repeated gene family and the similarity of many of the gene family members which are tandemly arrayed and clustered on linkage group B4 [22]. It would be interesting to determine the extent of LD around other tandemly repeated genes. On the other hand, recombination can be suppressed at an introgression event when parts of a distantly related genome are backcrossed into a common background as happens when plant breeding directs the selection of a gene from a wild relative into a cultivated background.

### Association of bruchid resistance and the APA locus

The data of the germplasm survey confirm that the APA family is responsible for bruchid resistance observed in this study as associations between the percentage adult emergence and the markers from the APA locus and even those flanking this locus were very significant. These results agree with the first evaluations of arcelin and the APA locus by Cardona et al. [29], Fory et al. [16], Kornegay et al. [19] and Osborn et al. [47]. More recent results of Zambre et al. [17] with transgenic tepary bean transformed with arcelin and of Goossens et al. [6] with artificial arcelin-containing seeds question the role of arcelin in resistance but these results may be due to the effect of heterologous expression or reduced concentration of the seed protein. Alternatively, other genes outside the APA locus may be needed in combination with arcelin for resistance to be expressed. Supporting this hypothesis Nishizawa et al. [20] found that bruchid resistance could be lost with backcrossing of arcelin alleles into a susceptible genotype.

Quantitative data on *Z. subfasciatus *egg laying and adult emergence across all the genotypes showed that these two traits were not correlated, therefore the mechanism of resistance was not based on any antibiosis at the seed coat level but rather on antibiosis to the development of the insect within the seed as was known previously. Despite this wild beans generally had fewer eggs laid per seed because the seed of these genotypes is so much smaller than cultivated beans. It would be interesting to explore this further especially in different sized common or lima beans as some differences in seed coat preferences have been noted [48] and both female fecundity and the numbers of insects emerging per seed in susceptible bean lines have upper limits [49].

Meanwhile, the percentage adult emergence was correlated with the number of adults in the cultivated resistant genotypes but not in the wild resistant genotypes perhaps because of an association with the number of eggs laid or because of variability for level of resistance found in the wild accessions. It was notable that the most useful APA variants in our study were Arc1 in the cultivated background and Arc1, Arc4, Arc5 and Arc7 in the wild background and this is consistent with results from previous studies [19]. Genetic background effect from wild germplasm background was also observed by Cardona and Kornegay [2] for the first five arcelin alleles and in the transgenic testing of arcelin variants described above. Arc1 has been used to a greater extent in plant breeders' selections as it provided higher resistance in a wide array of cultivar types of different seed sizes or colors compared to Arc5 or the other wild-derived alleles.

Arc2, Arc3 and Arc4, on the other hand, have not been widely used for introgression, so creation and analysis of cultivated genotypes with these alleles would be of interest with the provision that they are compared to Arc1 or Arc5 isolines. Finally, some resistant genotypes had very few adults emerge before 50 days after infestation but then had a few emerge in the period from 50 to 70 days after infestation. This may indicate that in resistant genotypes, especially those with weaker arcelin alleles, emergence may be delayed but a few adults do develop to maturity indicating the effect of antibiosis over the extended lifestages of the insect. It may be useful to seek recombinants between different Arc alleles to determine if various members of the APA family are epistatically influencing the expression of bruchid resistance.

## Conclusions

The APA locus was found to be important in providing insecticidal properties to common bean seed under attack by the bruchid species *Z. fasciatus *and to be easily diagnosed by several closely-associated microsatellite markers. Moderate LD was found within the APA locus but not beyond it. Other regions throughout the genome with significant LD were perhaps related to domestication genes. In this sense the genetic tools developed in this study are useful for further work on introgression of Arc alleles from the wild to common bean cultivars and for dissecting the role of different members of the APA locus on bruchid resistance. The RAZ and GG lines were also found to be useful for characterizing introgression of bruchid resistance into a cultivated background.

## Authors' contributions

MWB conceived of the study together with CC and wrote the paper. SP evaluated insect resistance in bruchid assays and allele diversity. LMD analyzed the datasets. HFB carried out molecular marker confirmation. Both CC and MWB helped design the experiments and advised on bruchid and marker analysis, respectively. All authors read and approved the final manuscript.
